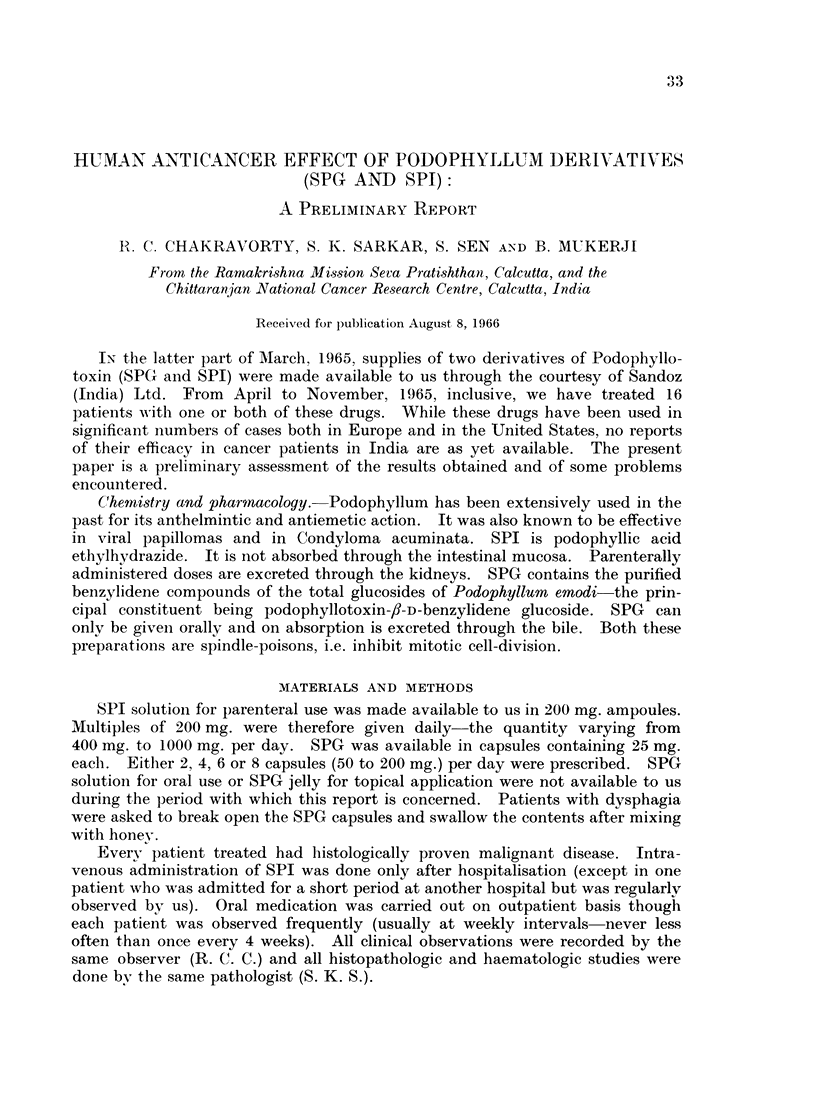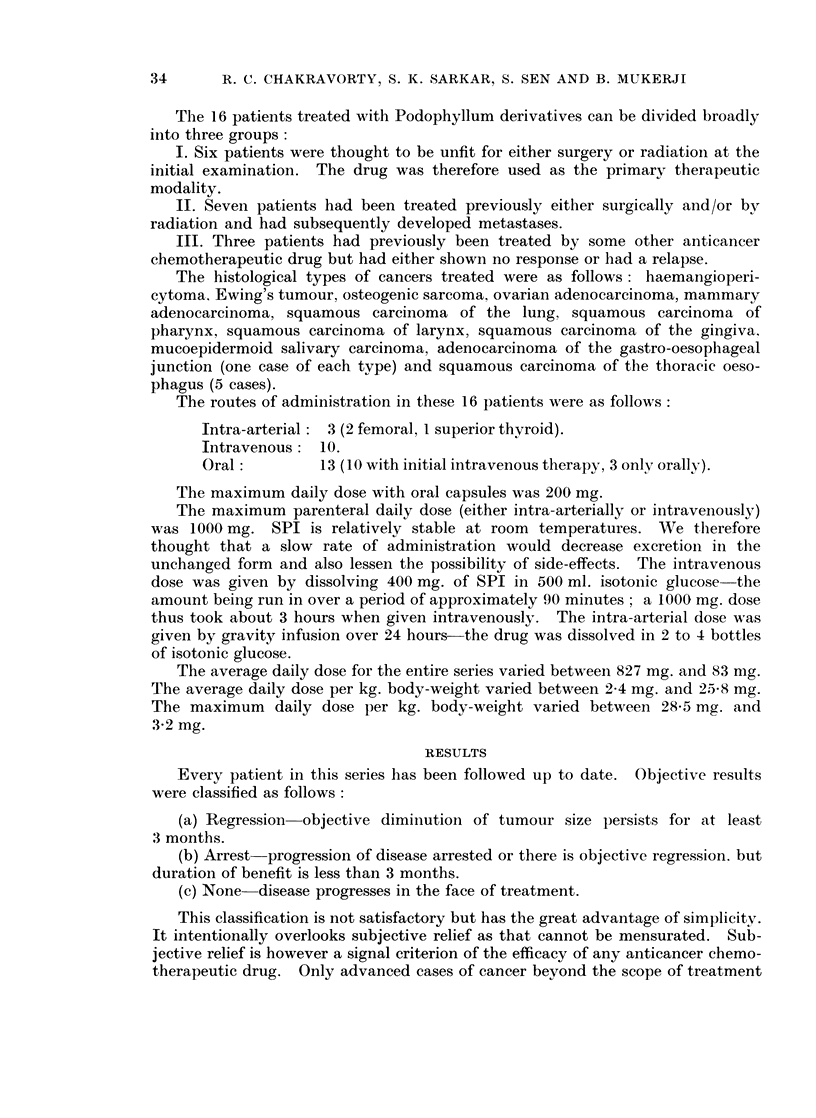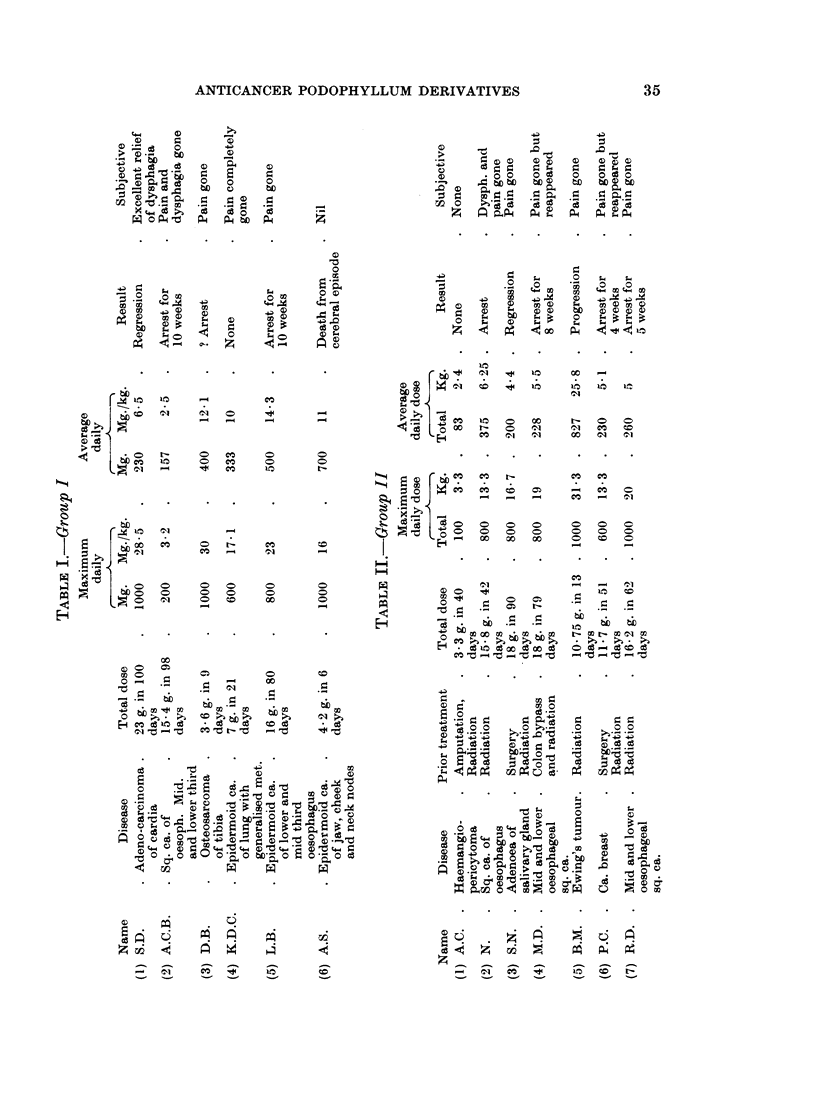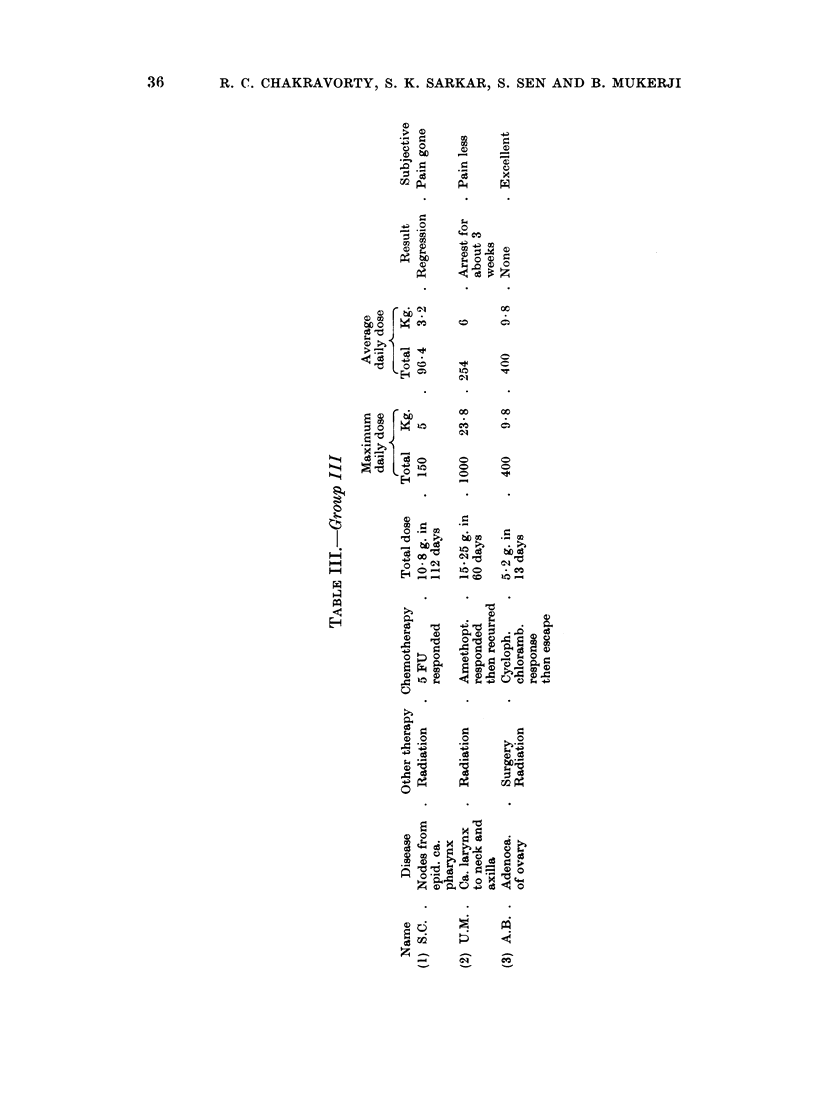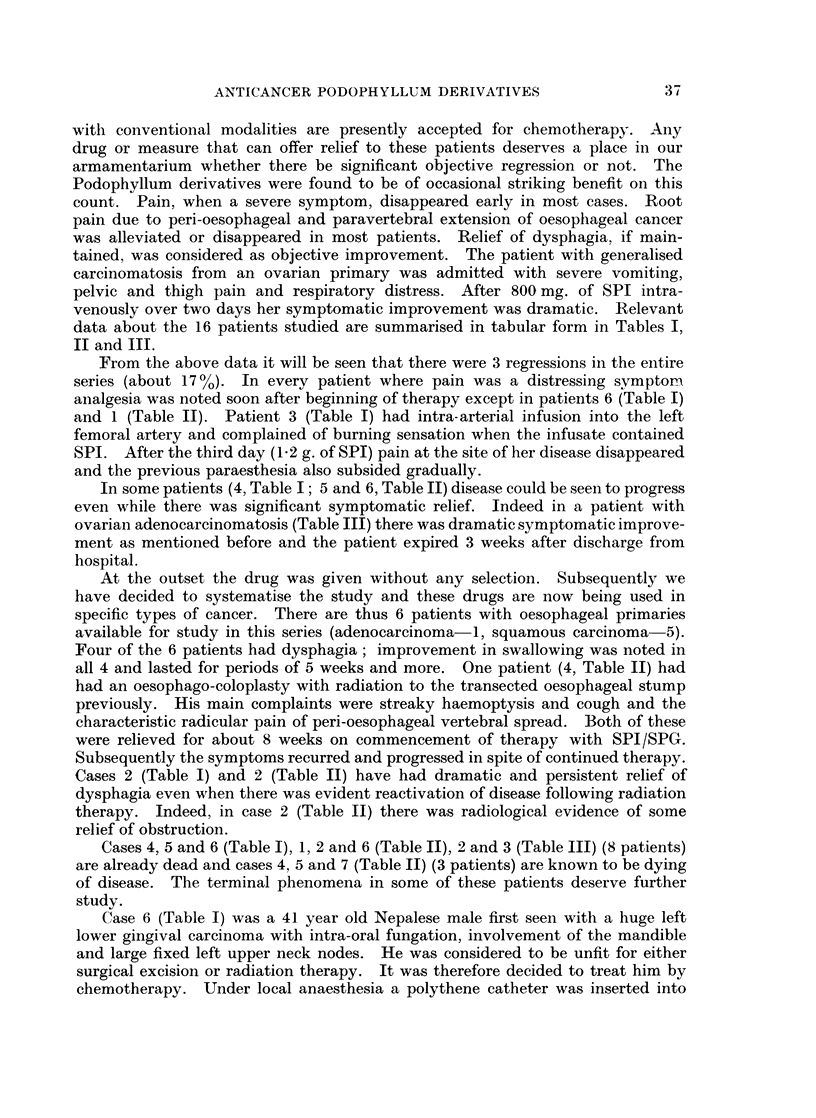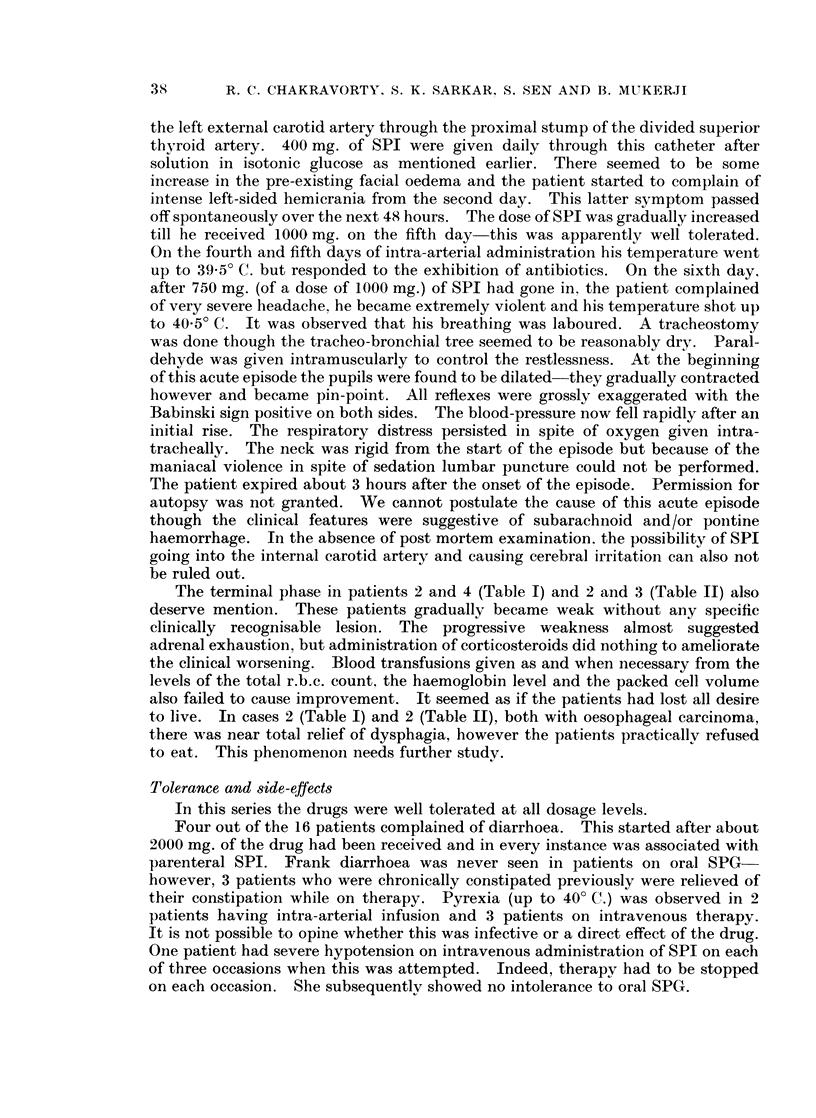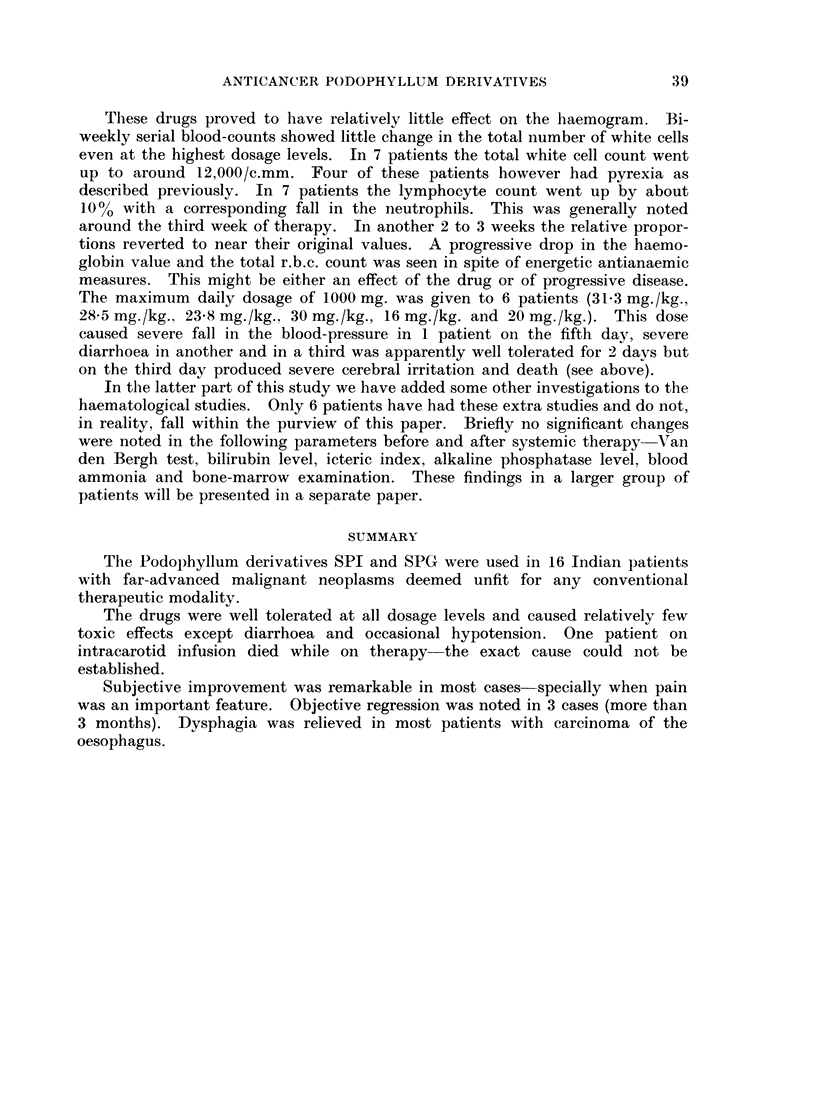# Human anticancer effect of podophyllum derivatives (SPG and SPI): a preliminary report.

**DOI:** 10.1038/bjc.1967.5

**Published:** 1967-03

**Authors:** R. C. Chakravorty, S. K. Sarkar, S. Sen, B. Mukerji


### HUMAN ANTICANCER EFFECT OF PODOPHYLLUM DERIVATIVES (SPG AND SPI): A PRELIMINARY REPORT

R. C. CHAKRAVORTY, S. K. SARKAR, S. SEN AND B. MUKERJI

*From the Ramakrishna Mission Seva Pratishthan, Calcutta, and the Chittaranjan National Cancer Research Centre, Calcutta, India*

Received for publication August 8, 1966

IN the latter part of March, 1965, supplies of two derivatives of Podophyllotoxin (SPG and SPI) were made available to us through the courtesy of Sandoz (India) Ltd. From April to November, 1965, inclusive, we have treated 16 patients with one or both of these drugs. While these drugs have been used in significant numbers of cases both in Europe and in the United States, no reports of their efficacy in cancer patients in India are as yet available. The present paper is a preliminary assessment of the results obtained and of some problems encountered.

*Chemistry and pharmacology.*—Podophyllum has been extensively used in the past for its anthelmintic and antiemetic action. It was also known to be effective in viral papillomas and in Condyloma acuminata. SPI is podophyllic acid ethylhydrazide. It is not absorbed through the intestinal mucosa. Parenterally administered doses are excreted through the kidneys. SPG contains the purified benzylidene compounds of the total glucosides of *Podophyllum emodi*—the principal constituent being podophyllotoxin-$\beta$-D-benzylidene glucoside. SPG can only be given orally and on absorption is excreted through the bile. Both these preparations are spindle-poisons, i.e. inhibit mitotic cell-division.

### MATERIALS AND METHODS

SPI solution for parenteral use was made available to us in 200 mg. ampoules. Multiples of 200 mg. were therefore given daily—the quantity varying from 400 mg. to 1000 mg. per day. SPG was available in capsules containing 25 mg. each. Either 2, 4, 6 or 8 capsules (50 to 200 mg.) per day were prescribed. SPG solution for oral use or SPG jelly for topical application were not available to us during the period with which this report is concerned. Patients with dysphagia were asked to break open the SPG capsules and swallow the contents after mixing with honey.

Every patient treated had histologically proven malignant disease. Intravenous administration of SPI was done only after hospitalisation (except in one patient who was admitted for a short period at another hospital but was regularly observed by us). Oral medication was carried out on outpatient basis though each patient was observed frequently (usually at weekly intervals—never less often than once every 4 weeks). All clinical observations were recorded by the same observer (R. C. C.) and all histopathologic and haematologic studies were done by the same pathologist (S. K. S.).

The 16 patients treated with Podophyllum derivatives can be divided broadly into three groups :

I. Six patients were thought to be unfit for either surgery or radiation at the initial examination. The drug was therefore used as the primary therapeutic modality.

II. Seven patients had been treated previously either surgically and/or by radiation and had subsequently developed metastases.

III. Three patients had previously been treated by some other anticancer chemotherapeutic drug but had either shown no response or had a relapse.

The histological types of cancers treated were as follows : haemangiopericytoma, Ewing's tumour, osteogenic sarcoma, ovarian adenocarcinoma, mammary adenocarcinoma, squamous carcinoma of the lung, squamous carcinoma of pharynx, squamous carcinoma of larynx, squamous carcinoma of the gingiva, mucoepidermoid salivary carcinoma, adenocarcinoma of the gastro-oesophageal junction (one case of each type) and squamous carcinoma of the thoracic oesophagus (5 cases).

The routes of administration in these 16 patients were as follows :

| | |
|---|---|
| Intra-arterial : | 3 (2 femoral, 1 superior thyroid). |
| Intravenous : | 10. |
| Oral : | 13 (10 with initial intravenous therapy, 3 only orally). |

The maximum daily dose with oral capsules was 200 mg.

The maximum parenteral daily dose (either intra-arterially or intravenously) was 1000 mg. SPI is relatively stable at room temperatures. We therefore thought that a slow rate of administration would decrease excretion in the unchanged form and also lessen the possibility of side-effects. The intravenous dose was given by dissolving 400 mg. of SPI in 500 ml. isotonic glucose—the amount being run in over a period of approximately 90 minutes ; a 1000 mg. dose thus took about 3 hours when given intravenously. The intra-arterial dose was given by gravity infusion over 24 hours—the drug was dissolved in 2 to 4 bottles of isotonic glucose.

The average daily dose for the entire series varied between 827 mg. and 83 mg. The average daily dose per kg. body-weight varied between 2·4 mg. and 25·8 mg. The maximum daily dose per kg. body-weight varied between 28·5 mg. and 3·2 mg.

### RESULTS

Every patient in this series has been followed up to date. Objective results were classified as follows :

(a) Regression—objective diminution of tumour size persists for at least 3 months.

(b) Arrest—progression of disease arrested or there is objective regression, but duration of benefit is less than 3 months.

(c) None—disease progresses in the face of treatment.

This classification is not satisfactory but has the great advantage of simplicity. It intentionally overlooks subjective relief as that cannot be mensurated. Subjective relief is however a signal criterion of the efficacy of any anticancer chemotherapeutic drug. Only advanced cases of cancer beyond the scope of treatment

TABLE I.—*Group I*

| | Name | Disease | Total dose | Maximum daily Mg. | Maximum daily Mg./kg. | Average daily Mg. | Average daily Mg./kg. | Result | Subjective |
|---|---|---|---|---|---|---|---|---|---|
| (1) | S.D. | Adeno-carcinoma of cardia | 23 g. in 100 days | 1000 | 28·5 | 230 | 6·5 | Regression | Excellent relief of dysphagia |
| (2) | A.C.B. | Sq. ca. of oesoph. Mid. and lower third | 15·4 g. in 98 days | 200 | 3·2 | 157 | 2·5 | Arrest for 10 weeks | Pain and dysphagia gone |
| (3) | D.B. | Osteosarcoma of tibia | 3·6 g. in 9 days | 1000 | 30 | 400 | 12·1 | ? Arrest | Pain gone |
| (4) | K.D.C. | Epidermoid ca. of lung with generalised met. | 7 g. in 21 days | 600 | 17·1 | 333 | 10 | None | Pain completely gone |
| (5) | L.B. | Epidermoid ca. of lower and mid third oesophagus | 16 g. in 80 days | 800 | 23 | 500 | 14·3 | Arrest for 10 weeks | Pain gone |
| (6) | A.S. | Epidermoid ca. of jaw, cheek and neck nodes | 4·2 g. in 6 days | 1000 | 16 | 700 | 11 | Death from cerebral episode | Nil |

TABLE II.—*Group II*

| | Name | Disease | Prior treatment | Total dose | Maximum daily dose Total | Maximum daily dose Kg. | Average daily dose Total | Average daily dose Kg. | Result | Subjective |
|---|---|---|---|---|---|---|---|---|---|---|
| (1) | A.C. | Haemangio-pericytoma | Amputation, Radiation | 3·3 g. in 40 days | 100 | 3·3 | 83 | 2·4 | None | None |
| (2) | N. | Sq. ca. of oesophagus | Radiation | 15·8 g. in 42 days | 800 | 13·3 | 375 | 6·25 | Arrest | Dysph. and pain gone |
| (3) | S.N. | Adenoea of salivary gland | Surgery Radiation | 18 g. in 90 days | 800 | 16·7 | 200 | 4·4 | Regression | Pain gone |
| (4) | M.D. | Mid and lower oesophageal sq. ca. | Colon bypass and radiation | 18 g. in 79 days | 800 | 19 | 228 | 5·5 | Arrest for 8 weeks | Pain gone but reappeared |
| (5) | B.M. | Ewing's tumour | Radiation | 10·75 g. in 13 days | 1000 | 31·3 | 827 | 25·8 | Progression | Pain gone |
| (6) | P.C. | Ca. breast | Surgery Radiation | 11·7 g. in 51 days | 600 | 13·3 | 230 | 5·1 | Arrest for 4 weeks | Pain gone but reappeared |
| (7) | R.D. | Mid and lower oesophageal sq. ca. | Radiation | 16·2 g. in 62 days | 1000 | 20 | 260 | 5 | Arrest for 5 weeks | Pain gone |

TABLE III.—*Group III*

| Name | Disease | Other therapy | Chemotherapy | Total dose | Maximum daily dose | | Average daily dose | | Result | Subjective |
|---|---|---|---|---|---|---|---|---|---|---|
| | | | | | Total | Kg. | Total | Kg. | | |
| (1) S.C. | Nodes from epid. ca. pharynx | Radiation | 5 FU responded | 10·8 g. in 112 days | 150 | 5 | 96·4 | 3·2 | Regression | Pain gone |
| (2) U.M. | Ca. larynx to neck and axilla | Radiation | Amethopt. responded then recurred | 15·25 g. in 60 days | 1000 | 23·8 | 254 | 6 | Arrest for about 3 weeks | Pain less |
| (3) A.B. | Adenoca. of ovary | Surgery Radiation | Cycloph. chloramb. response then escape | 5·2 g. in 13 days | 400 | 9·8 | 400 | 9·8 | None | Excellent |

with conventional modalities are presently accepted for chemotherapy. Any drug or measure that can offer relief to these patients deserves a place in our armamentarium whether there be significant objective regression or not. The Podophyllum derivatives were found to be of occasional striking benefit on this count. Pain, when a severe symptom, disappeared early in most cases. Root pain due to peri-oesophageal and paravertebral extension of oesophageal cancer was alleviated or disappeared in most patients. Relief of dysphagia, if maintained, was considered as objective improvement. The patient with generalised carcinomatosis from an ovarian primary was admitted with severe vomiting, pelvic and thigh pain and respiratory distress. After 800 mg. of SPI intravenously over two days her symptomatic improvement was dramatic. Relevant data about the 16 patients studied are summarised in tabular form in Tables I, II and III.

From the above data it will be seen that there were 3 regressions in the entire series (about 17%). In every patient where pain was a distressing symptom analgesia was noted soon after beginning of therapy except in patients 6 (Table I) and 1 (Table II). Patient 3 (Table I) had intra-arterial infusion into the left femoral artery and complained of burning sensation when the infusate contained SPI. After the third day (1·2 g. of SPI) pain at the site of her disease disappeared and the previous paraesthesia also subsided gradually.

In some patients (4, Table I; 5 and 6, Table II) disease could be seen to progress even while there was significant symptomatic relief. Indeed in a patient with ovarian adenocarcinomatosis (Table III) there was dramatic symptomatic improvement as mentioned before and the patient expired 3 weeks after discharge from hospital.

At the outset the drug was given without any selection. Subsequently we have decided to systematise the study and these drugs are now being used in specific types of cancer. There are thus 6 patients with oesophageal primaries available for study in this series (adenocarcinoma—1, squamous carcinoma—5). Four of the 6 patients had dysphagia; improvement in swallowing was noted in all 4 and lasted for periods of 5 weeks and more. One patient (4, Table II) had had an oesophago-coloplasty with radiation to the transected oesophageal stump previously. His main complaints were streaky haemoptysis and cough and the characteristic radicular pain of peri-oesophageal vertebral spread. Both of these were relieved for about 8 weeks on commencement of therapy with SPI/SPG. Subsequently the symptoms recurred and progressed in spite of continued therapy. Cases 2 (Table I) and 2 (Table II) have had dramatic and persistent relief of dysphagia even when there was evident reactivation of disease following radiation therapy. Indeed, in case 2 (Table II) there was radiological evidence of some relief of obstruction.

Cases 4, 5 and 6 (Table I), 1, 2 and 6 (Table II), 2 and 3 (Table III) (8 patients) are already dead and cases 4, 5 and 7 (Table II) (3 patients) are known to be dying of disease. The terminal phenomena in some of these patients deserve further study.

Case 6 (Table I) was a 41 year old Nepalese male first seen with a huge left lower gingival carcinoma with intra-oral fungation, involvement of the mandible and large fixed left upper neck nodes. He was considered to be unfit for either surgical excision or radiation therapy. It was therefore decided to treat him by chemotherapy. Under local anaesthesia a polythene catheter was inserted into the left external carotid artery through the proximal stump of the divided superior thyroid artery. 400 mg. of SPI were given daily through this catheter after solution in isotonic glucose as mentioned earlier. There seemed to be some increase in the pre-existing facial oedema and the patient started to complain of intense left-sided hemicrania from the second day. This latter symptom passed off spontaneously over the next 48 hours. The dose of SPI was gradually increased till he received 1000 mg. on the fifth day—this was apparently well tolerated. On the fourth and fifth days of intra-arterial administration his temperature went up to 39·5° C. but responded to the exhibition of antibiotics. On the sixth day, after 750 mg. (of a dose of 1000 mg.) of SPI had gone in, the patient complained of very severe headache, he became extremely violent and his temperature shot up to 40·5° C. It was observed that his breathing was laboured. A tracheostomy was done though the tracheo-bronchial tree seemed to be reasonably dry. Paraldehyde was given intramuscularly to control the restlessness. At the beginning of this acute episode the pupils were found to be dilated—they gradually contracted however and became pin-point. All reflexes were grossly exaggerated with the Babinski sign positive on both sides. The blood-pressure now fell rapidly after an initial rise. The respiratory distress persisted in spite of oxygen given intratracheally. The neck was rigid from the start of the episode but because of the maniacal violence in spite of sedation lumbar puncture could not be performed. The patient expired about 3 hours after the onset of the episode. Permission for autopsy was not granted. We cannot postulate the cause of this acute episode though the clinical features were suggestive of subarachnoid and/or pontine haemorrhage. In the absence of post mortem examination, the possibility of SPI going into the internal carotid artery and causing cerebral irritation can also not be ruled out.

The terminal phase in patients 2 and 4 (Table I) and 2 and 3 (Table II) also deserve mention. These patients gradually became weak without any specific clinically recognisable lesion. The progressive weakness almost suggested adrenal exhaustion, but administration of corticosteroids did nothing to ameliorate the clinical worsening. Blood transfusions given as and when necessary from the levels of the total r.b.c. count, the haemoglobin level and the packed cell volume also failed to cause improvement. It seemed as if the patients had lost all desire to live. In cases 2 (Table I) and 2 (Table II), both with oesophageal carcinoma, there was near total relief of dysphagia, however the patients practically refused to eat. This phenomenon needs further study.

*Tolerance and side-effects*

In this series the drugs were well tolerated at all dosage levels.

Four out of the 16 patients complained of diarrhoea. This started after about 2000 mg. of the drug had been received and in every instance was associated with parenteral SPI. Frank diarrhoea was never seen in patients on oral SPG—however, 3 patients who were chronically constipated previously were relieved of their constipation while on therapy. Pyrexia (up to 40° C.) was observed in 2 patients having intra-arterial infusion and 3 patients on intravenous therapy. It is not possible to opine whether this was infective or a direct effect of the drug. One patient had severe hypotension on intravenous administration of SPI on each of three occasions when this was attempted. Indeed, therapy had to be stopped on each occasion. She subsequently showed no intolerance to oral SPG.

These drugs proved to have relatively little effect on the haemogram. Biweekly serial blood-counts showed little change in the total number of white cells even at the highest dosage levels. In 7 patients the total white cell count went up to around 12,000/c.mm. Four of these patients however had pyrexia as described previously. In 7 patients the lymphocyte count went up by about 10% with a corresponding fall in the neutrophils. This was generally noted around the third week of therapy. In another 2 to 3 weeks the relative proportions reverted to near their original values. A progressive drop in the haemoglobin value and the total r.b.c. count was seen in spite of energetic antianaemic measures. This might be either an effect of the drug or of progressive disease. The maximum daily dosage of 1000 mg. was given to 6 patients (31·3 mg./kg., 28·5 mg./kg., 23·8 mg./kg., 30 mg./kg., 16 mg./kg. and 20 mg./kg.). This dose caused severe fall in the blood-pressure in 1 patient on the fifth day, severe diarrhoea in another and in a third was apparently well tolerated for 2 days but on the third day produced severe cerebral irritation and death (see above).

In the latter part of this study we have added some other investigations to the haematological studies. Only 6 patients have had these extra studies and do not, in reality, fall within the purview of this paper. Briefly no significant changes were noted in the following parameters before and after systemic therapy—Van den Bergh test, bilirubin level, icteric index, alkaline phosphatase level, blood ammonia and bone-marrow examination. These findings in a larger group of patients will be presented in a separate paper.

### SUMMARY

The Podophyllum derivatives SPI and SPG were used in 16 Indian patients with far-advanced malignant neoplasms deemed unfit for any conventional therapeutic modality.

The drugs were well tolerated at all dosage levels and caused relatively few toxic effects except diarrhoea and occasional hypotension. One patient on intracarotid infusion died while on therapy—the exact cause could not be established.

Subjective improvement was remarkable in most cases—specially when pain was an important feature. Objective regression was noted in 3 cases (more than 3 months). Dysphagia was relieved in most patients with carcinoma of the oesophagus.